# The (im-)moral scientist? Measurement and framing effects shape the association between scientists and immorality

**DOI:** 10.1371/journal.pone.0274379

**Published:** 2022-10-03

**Authors:** Bastiaan T. Rutjens, Esther Niehoff, Steven J. Heine

**Affiliations:** 1 Department of Psychology, University of Amsterdam, Amsterdam, the Netherlands; 2 Department of Psychology, University of British Columbia, Vancouver, Canada; Coventry University, UNITED KINGDOM

## Abstract

Recent years have not only seen growing public distrust in science, but also in the people conducting science. Yet, attitudes toward scientists remain largely unexplored, and the limited body of literature that exists points to an interesting ambivalence. While survey data suggest scientists to be positively evaluated (e.g., respected and trusted), research has found scientists to be perceived as capable of immoral behavior. We report two experiments aimed at identifying what contributes to this ambivalence through systematic investigations of stereotypical perceptions of scientists. In these studies, we particularly focus on two potential sources of inconsistencies in previous work: divergent operationalizations of morality (measurement effects), and different specifications of the broad group of scientists (framing effects). Results show that scientists are generally perceived as more likely to violate binding as opposed to individualizing moral foundations, and that they deviate from control groups more strongly on the latter. The extent to which different morality measures reflect the differentiation between binding and individualizing moral foundations at least partially accounts for previous contradictory findings. Moreover, the results indicate large variation in perceptions of different types of scientists: people hold more positive attitudes toward university-affiliated scientists as compared to industry-affiliated scientists, with perceptions of the ‘typical scientist’ more closely resembling the latter. Taken together, the findings have important academic ramifications for science skepticism, morality, and stereotyping research as well as valuable practical implications for successful science communication.

## Introduction

Increases in science skepticism among the public represent one of the key societal challenges of the 21st century. Never has this been clearer than in 2020. For the past decade, rising ocean temperatures and increasing numbers of measles outbreaks have had scientists worried about climate change denial and vaccination skepticism [1–3]. Yet, the COVID-19 pandemic is an unprecedented demonstration of the immediate threat that science skepticism constitutes to society–whether it is skeptics’ neglect of social distancing measures, their protest against a yet to be developed vaccine (as of 2020), or plain disbelief in the existence of the virus [4–6]. Critically, these behaviors and beliefs often seem to be accompanied by discussions surrounding the trustworthiness of the expert scientists in charge of countries’ responses to the pandemic (such as Dr. Fauci in the United States, Dr. Drosten in Germany, or Dr. Van Dissel in the Netherlands). Given long-standing cultural archetypes of the evil scientist, widely publicized cases of scientific fraud, and increasingly popular theories of biased agenda and conspiracy [7–9], this skepticism toward scientists is not new. However, while considerable effort has been put in analyzing the predictors of science skepticism (see Rutjens et al. [10] for a recent overview), social perceptions of the people conducting science as well as the influence of these perceptions on science distrust have received little attention. Furthering knowledge on how the public evaluates scientists has the potential to provide vital insights into effective science communication, with direct implications for pressing social and political issues, such as climate change mitigation or containment of the COVID-19 pandemic.

### Skepticism toward science and scientists

In order to understand how scientists are evaluated, it is insightful to first turn to public attitudes toward science in general. Much existing research on science skepticism has focused on individual differences in worldviews and ideologies. Low faith in science and low willingness to support science (through the allocation of monetary resources) were found to be best predicted by belief systems ranging from religious orthodoxy to spirituality, thereby providing further support for the well-established oppositional relation between science and religion [11, 12]. Political conservatism is another key predictor of science skepticism, in particular toward climate science [11, 13, 14]. Additionally, many scientific debates, for example about genetically modified organisms (GMO), speak to people’s moral views about the world. In particular, concerns about purity and naturalness seem to have the potential to interfere with evaluations of scientific evidence if that evidence poses a threat to the individual’s moral values [11, 15]. In line with that finding, people have been found to selectively put their trust in scientific findings that are in line with their moral values [16]. Likewise, the degree to which people deemed a scientific hypothesis to be morally offensive negatively predicts the perceived credibility and acceptance of that hypothesis [17]. Moral values also play a role in research on specific domains of science skepticism, with data suggesting that both vaccine skepticism [18] and GMO resistance [11, 15] are at least partially driven by disgust and concerns about moral purity.

There is also evidence suggesting that science distrust is motivated by moral concerns about scientists. Rutjens and Heine [19] systematically investigated whether scientists are perceived as immoral across ten studies. Utilizing Moral Foundations Theory [20] as a framework, they found that participants perceived scientists as likely to commit breaches against binding moral foundations (i.e., loyalty, authority, purity), particularly against purity norms, yet not against individualizing foundations (i.e., care, fairness). Moreover, participants judged scientists to prioritize knowledge gain over morality, to lack human emotions, and even to be potentially dangerous. These findings are in line with other work on public perceptions of scientists, such as analyses of the image of scientists in Western literature and film. Tintori [21], for example, concluded that scientists are often portrayed as “isolated in their ivory tower, focused on their work, crazy, evil and dangerous” (p. 18). Others have suggested that scientists are perceived as having an unhealthy ambition in the pursuit of (forbidden) knowledge, which makes them potentially mad and dangerous [9, 22, 23]. Particularly this last idea is also reflected in survey data, which indicated that close to two thirds of EU citizens believe science to sometimes interfere with people’s sense of ethics, with half even believing scientists to “have a power that makes them dangerous” [24, see also 25]. These concerns about the morality of scientists are especially striking because other research finds that scientists are perceived as smart, highly competent, eminently respected, trusted, and even liked [26, 27]. In short, people seem to have quite ambivalent attitudes towards scientists.

### Potential sources of contradictory findings

These opposing attitudes suggest there are hidden complexities in how scientists are evaluated that require further exploration. Complexities may lie both in how morality is conceptualized and operationalized, and in how people imagine what constitutes a scientist. Regarding morality, most previous studies have focused on Moral Foundation Theory’s categorization of the five moral foundations of care, fairness, loyalty, authority, and purity [20]. Yet, other theories of morality have employed the Stereotype Content Model (SCM), which proposes that people assess others along two primary dimensions of warmth and competence [28], by conceptualizing morality as one of two components of warmth (the other being sociability [29, 30]). Applying the SCM to scientists has revealed that scientists tend to be perceived as high in competence, but low in warmth [27]. Yet, an analysis that differentiates scientists’ perceived warmth into their perceived morality and sociability has yet to be conducted. Given the ambivalence in people’s views of scientists, distinguishing between the two components of warmth may be illuminating. Leach and colleagues [31] for example, applied this distinction in a study on anti-Semitic attitudes, showing that participants from the Russian Federation tended to perceive Jewish people as high in sociability but low in morality. Had only the overarching factor of warmth been examined, this important moral dimension of stereotypical perceptions of Jewish people amongst Russian participants would have likely been missed.

Second, the ambivalence that people hold towards scientists may be a product of how scientists have been defined or framed in the respective studies. Existing work has focused on perceptions of the “typical scientist” or of the broad group of scientists in general [19]. This approach may have neglected any nuances between different types of scientists, and it is quite likely that participants brought to mind quite different prototypes of scientists, thus adding much noise to the data. The present research sought to correct the limitations of past research by including different measures of morality and by asking people about different types of scientists, including self-generated examples.

### Overview of studies

We conducted two studies aimed at investigating stereotypical perceptions of scientists, with a particular focus on perceptions of morality. Study 1 (conducted in March 2018) built upon insights from previous work that revealed associations between scientists and immorality [19], yet made use of more focused and well-established measures and materials. More specifically, we included two different measures of morality and employed different framings of scientists. In Study 2 (conducted in December 2018), we sought to replicate Study 1’s findings in another sample, using other control groups, a different design (between-subjects instead of within-subjects), as well as a different framing of scientists (a bottom-up approach of self-generated examples). In addition, Study 2 also explored the relationship between science skeptical attitudes and their relationship with perceived skeptical attitudes held by scientists as well as perceptions of scientists. All measures, manipulations, and data exclusions are reported.

## Study 1

In Study 1, we aimed to establish how scientists are perceived in terms of morality, sociability, and competence. We compared these perceptions to those of two opposing control groups, namely atheists and religious people. Moreover, we were interested in whether different types of scientists (i.e., scientists working in academia or in industry) are perceived differently. Therefore, next to the typical scientists and the control groups, we included assessments on two specific framings of scientists.

### Method

#### Participants

One hundred and fifty participants were recruited from the student pool at University of Amsterdam. Ethics approval was obtained at the first author’s host institution (2018-SP-8787). All participants provided written consent before participating in the study. After exclusion of 4 participants due to incomplete responses and of 6 participants due to a failed manipulation check, data from 140 participants were used in the analyses. The average age was 21.13 years (*SD* = 4.02), and 77.40% identified as women. A sensitivity analysis conducted with G*Power software [32] indicated that this sample size was sufficient for detecting small effects of size f = 0.14 or larger.

#### Procedure and materials

Participants were asked to make judgments about scientists and two control groups, namely atheists and religious people. To that end, participants were introduced to three fictional characters: a scientist, an atheist, and a religious person, whom they rated in terms of morality (honest, sincere, trustworthy), sociability (likeable, warm, friendly), and competence (competent, intelligent, skillful) based on Leach and colleagues’ [30] three dimensions of stereotype content. Moreover, a two-item general moral character scale (good-bad, good-bad moral standards) was included [33].

Next, participants were randomly allocated to one of two conditions and introduced to yet another character: one half of participants was introduced to a scientist working for a university, while the other half was introduced to a scientist working in the pharmaceutical industry. They were then asked to make the same and additional judgments about this character as before. Participants were also asked to indicate the extent to which they believed the introduced characters cared about the following concepts: pursuing one’s curiosity [19], pursuing one’s desire, as well as the five moral foundations of harm/care, justice/fairness (here, honesty), loyalty, authority, and naturalness (as a proxy for purity) [20]. All responses were given on a Likert type scale ranging from 1 for strongly disagree to 7 for strongly agree.

## Results

First, we created scales for morality (honest, sincere, trustworthy; α = .88), sociability (likeable, warm, friendly; α = .91), and competence (competent, intelligent, skillful; α = .91) by calculating a mean across the three individual items. The two items of the immoral character scale were highly correlated (*r =* .74). Internal reliabilities for each group separately as well as zero-order correlations for all variables can be found in the S1 and S2 Tables. We then conducted univariate ANOVAs to test whether the groups differed on these variables, which were followed by post-hoc Tukey HSD tests. The results, along with means and standard deviations for all items (and scales) are presented in Table 1.

**Table 1:** Means and standard deviations for all groups and results of ANOVA and Tukey HSD tests.

	ANOVA		Tukey HSD				
	F	η^2^	1. Scientist	2. Atheist	3. Religious	4. University-S	5. Industry-S
Morality (F)	33.85***	.190	5.10 (±0.87)^2^^,^^4^^,^^5^	4.76 (±0.76)*	4.87 (±0.93)	5.74 (±0.84)***	4.12 (±1.12)***
Honest	22.07***	.134	5.12 (±0.98)^2^^,^^4^^,^^5^	4.78 (±0.87)**	4.90 (±1.12)	5.69 (±0.95)***	4.23 (±1.19)***
Sincere	26.62***	.157	4.97 (±1.00)^4^^,^^5^	4.86 (±0.91)	4.80 (±1.05)	5.81 (±0.96)***	4.10 (±1.16)***
Trustworthy	32.31***	.184	5.19 (±0.96)^2^^,^^3^^,^^4^^,^^5^	4.65 (±0.90)***	4.89 (±1.06)*	5.72 (±0.94)**	4.03 (±1.21)***
Sociability (F)	18.00***	.111	4.54 (±0.83)^3^^,^^4^	4.71 (±0.83)	4.98 (±0.83)***	5.38 (±0.90)***	4.42 (±0.86)
Likeable	10.87***	.071	4.61 (±0.87)^3^^,^^4^	4.75 (±0.91)	4.89 (±0.90)*	5.34 (±0.98)***	4.47 (±0.96)
Warm	21.39***	.129	4.38 (±0.96)^3^^,^^4^	4.59 (±0.91)	4.97 (±0.95)***	5.43 (±1.09)***	4.27 (±0.92)
Friendly	13.93***	.089	4.62 (± 0.89)^3^^,^^4^	4.78 (±0.91)	5.08 (±0.89)***	5.38 (±0.92)***	4.50 (±0.88)
Competence (F)	109.9***	.432	5.83 (±0.87)^2^^,^^3^^,^^4^	4.90 (±0.76)***	4.32 (±0.93)***	6.20 (±0.74)*	5.93 (±0.73)
Competent	56.67***	.281	5.60 (±1.06)^2^^,^^3^^,^^4^	4.95 (±0.88)***	4.35 (±0.92)***	6.05 (±0.93)**	5.73 (±0.95)
Intelligent	117.4***	.447	6.08 (±1.01)^2^^,^^3^	5.03 (±0.93)***	4.23 (±0.97)***	6.38 (±0.79)	6.16 (±0.76)
Skillful	89.49***	.381	5.82 (±0.98)^2^^,^^3^^,^^4^	4.73 (±0.85)***	4.39 (±0.90)***	6.16 (±0.83)*	5.85 (±0.85)
Immoral character (F)	12.49***	.080	3.08 (±1.10)^4^	3.20 (±0.94)	3.04 (±1.05)	2.30 (±1.27)***	3.45 (±1.20)
Bad	11.75***	.075	2.95 (±1.16)^4^^,^^5^	3.13 (±1.04)	2.97 (±1.10)	2.22 (±1.30)***	3.38 (±1.17)*
Bad standards	9.81***	.064	3.11 (±1.21)^4^	3.27 (±1.04)	3.11 (±1.23)	2.39 (±1.30)***	3.52 (±1.34)
Ind. foundations (F)	54.24***	.278	n/a	n/a	n/a	5.20 (±0.81)^5^	4.06 (±1.08)***
Harm/care	45.10***	.247	n/a	n/a	n/a	5.11 (±1.05)^5^	3.88 (±1.11)***
Honesty (Justice)	36.64***	.204	n/a	n/a	n/a	5.23 (±0.92)^5^	4.15 (±1.25)***
Bind. foundations (F)	1.61	.011	n/a	n/a	n/a	4.20 (±0.90)	4.02 (±0.95)
Loyalty	4.72*	.033	n/a	n/a	n/a	4.35 (±1.10)^5^	3.92 (±1.27)*
Authority	3.38	.020	n/a	n/a	n/a	3.95 (±1.16)^5^	4.36 (±1.15)*
Purity	4.76*	.032	n/a	n/a	n/a	4.26 (±1.46)^5^	3.74 (±1.36)*
Curiosity	32.21***	.187	n/a	n/a	n/a	6.30 (±0.86)^5^	5.40 (±1.02)***
Desire	5.57*	.042	n/a	n/a	n/a	5.19 (±1.22)^5^	5.64 (±0.92)*

*Note*. *N* = 140 (except condition 4 university-scientist: *n* = 74, and condition 5 industry-scientist: *n* = 65). Degrees of freedom for ANOVAs (above separation line): 4, 578. Degrees of freedom for ANOVAs (below separation line):

1, 141. Subscripts refer to significant differences compared to

^2^atheist

^3^religious person

^4^university-scientist, and

^5^industry-scientist.

*p < .05

**p < .01

***p < .001

Overall, we found the typical scientist to be perceived as more moral than an atheist (*p* = .012, Cohen’s *d* = 0.416), but not than a religious person (*p* = .174, *d* = 0.255); less social than a religious person (*p* < .001, *d* = 0.530), but not an atheist (*p* = .426, *d* = 0.205); and as more competent than both an atheist and religious person (*ps* < .001, *d*_*atheist*_ = 1.139, *d*_*religious*_ = 1.677). For sociability and competence, these findings also hold for the individual item level (i.e., we found the same pattern for all three items per scale). For morality, however, the observed differences seem to be mostly driven by a significant difference in trustworthiness (*p* < .001, *d* = 0.580) and honesty (*p* = .009, *d* = 0.367), while differences in sincerity were in the same direction, but not significant (*p* = .575, *d* = 0.115). No differences between target groups were found on the immoral character scale.

In the second part of Study 1, one half of participants repeated the same (and additional) ratings for a scientist working at a university and the other half for one working in the pharmaceutical industry. Compared to the typical scientist, participants perceived the university-scientist as overall more moral (*p* < .001, *d* = 0.748) and the industry-scientist as overall less moral (*p* < .001, *d* = 0.977). With regards to overall sociability, competence, and moral character, only the university-scientist showed significant differences to the typical scientist, with being perceived as overall more social (*p* < .001, *d* = 0.970), more competent (*p* = .014, *d* = 0.458), and as having a less immoral character (*p* < .001, *d* = 0.657). In line with findings about general morality, the industry-scientist was rated to have a somewhat more immoral character, (*p* = .065, *d* = 0.321). Thus, across the different scales, the university-scientist is persistently rated more positively than the industry-scientist. In terms of morality, the typical scientist is mid-way between both, while in terms of warmth and competence, scores of the typical scientist are closer to the negatively perceived industry-scientist than the university-scientist.

Additionally, the second part of Study 1 included rating the university- and the industry-scientist in terms of motivations to follow individualizing and binding moral foundations as well as their curiosity and desire. University-scientists were perceived as more motivated to follow individualizing moral foundations than industry-scientists (*p* < .001, *d* = 1.194)–a finding that holds for both the harm/care foundation (*p* < .001, *d* = 1.138) as well as the justice/honesty foundation (*p* = .001, *d* = 0.984). For binding moral foundations, the difference between the university-scientist and the industry-scientist was not significant for the total scale (*p* = .252, *d* = 0.195), however, differences were significant for all three of the individual binding foundations of loyalty, authority, and purity. Whereas the university-scientist was perceived as more motivated to comply with foundations of loyalty (*p* = .041, *d* = 0.362) and purity (*p* = .028, *d* = 0.369), the industry-scientist was perceived as more motivated to comply with the authority foundation (*p* = .037, *d* = 0.355). Similarly, for the last two motivations (following one’s own curiosity and satisfying one’s own desire), the university-scientist and industry-scientist differed in opposing directions: university-scientists were perceived as more motivated to follow their curiosity than industry-scientists (*p* < .001, *d* = 0.954), yet less motivated to satisfy their desire (*p* = .035, *d* = 0.416).

### Discussion

The results from Study 1 replicate previous findings on social perception, suggesting that scientists tend to be perceived as highly competent [27]. With regards to warmth, the findings are mixed: scientists seem to be perceived as low in sociability yet high in morality, which stresses the importance of differentiating between these two components. Whereas the finding on sociability is in line with extant research that found scientists to be low in warmth [27], the finding on morality seems to contradict previous studies which identified associations between scientists and immorality [19]. Yet, different measures were employed to assess morality in the respective studies. It appears that scientists score comparatively low on the (binding) moral foundations [19], however, are generally perceived as moral when assessing morality along with sociability as a component of warmth, following the three-dimensional approach to stereotype content. This approach describes morality as consisting of honesty, sincerity, and trustworthiness–a definition that aligns closely with only one of the moral foundations, namely the individualizing foundation of justice/fairness (Note that sociability does not seem to map onto any of the moral foundations; although one could make an argument for sociability being somewhat related to either the harm/care or the loyalty foundation.). Hence, there is convergence across these studies–scientists are perceived as relatively immoral *only* when morality is conceptualized in terms consistent with the binding foundations. Within-comparisons for the university-affiliated and industry-affiliated scientists, for whom both measures of morality were obtained, support this idea: both groups scored higher on morality assessed as one of three dimensions of stereotype content, as compared to the moral (particularly binding) foundations. Thus, these findings stress the importance of incorporating different measures to assess the construct of morality.

Second, Study 1 tested different framings of scientists and found that perceptions of scientists depend on the type of the scientist in question. The scientist framed to work at a university was overall perceived more positively (in terms of sociability, competence, and morality) than the typical scientist. The scientist framed to work in the pharmaceutical industry, however, was perceived as less moral than the typical scientist, yet equal in terms of sociability and competence. Thus, perceptions of the typical scientist were overall closer to those of the industry-scientist as compared to the university-scientist. It is important to note that our example of industry scientists—scientists working in the pharmaceutical industry—might not generalize to other domains of industry science. However, the main goal of this aspect of the study was to show how different framings of scientists may lead to different social evaluations. In this light, it is also worth pointing out that the study was conducted before the COVID-19 pandemic.

This may indicate that people conceptualize scientists more as commercially working people who pursue economic benefits than employees of the public sector pursuing knowledge. Support for this differentiation comes from the finding that university-scientists were perceived as more curious than industry-scientists, while industry-scientists were perceived as more eager to satisfy their desire than university-scientists. These findings are consistent with findings by McCright and colleagues [34], who showed that attitudes toward science differed for what they coined impact-science (i.e., science that identifies environmental and public health impacts of economic production) as opposed to production-science (i.e., science that provides new inventions or innovations for economic production). Our descriptions of the university- and the industry-scientists may be playing at a similar differentiation, with university-scientists more likely conducting impact-science and industry-scientists more likely performing production-science.

Lastly, Study 1 finds that scientists are perceived as more moral than atheists (but not religious people) and less social than religious people (but not atheists). However, it is unclear whether these patterns are driven more by people’s views towards scientists or towards atheists and religious people, particularly given previous work suggesting people hold strong attitudes–negative and positive, respectively–toward atheists and religious people [35, 36]. To disentangle these effects, Study 2 included different—more neutral—control groups. Moreover, Study 2 moved beyond classical measures of social perception by also asking participants to make assessments about scientists’ and other groups’ attitudes toward controversially debated topics of science.

## Study 2

In Study 2, we included three new control groups and collected responses on similar measures as in Study 1. In addition, we asked participants to evaluate specific (self-generated) prominent scientists, as well as to evaluate contentious science topics that spark controversies amongst the public.

### Method

#### Participants

Ethics approval was obtained at the first author’s host institution (2018-SP-8787). All participants provided written consent before participating in the study. We collected data from 273 American participants via Amazon Mechanical Turk. Due to incomplete responses, data of 26 participants had to be excluded, resulting in a final sample size of 247. The average age was 40.63 years (*SD* = 10.44), with 51.82% identifying as female. A sensitivity analysis conducted with G*Power software [32] indicated that this sample size was sufficient for detecting small effects of size f = 0.14 or larger.

#### Procedure and materials

Participants were first randomly assigned to one of two conditions: A scientist condition or a novelist condition. Our aim was to use a control occupational category that 1) was different from the categories used in Study 1 and 2) shares some similarities with the scientist category (e.g., engaging in intellectually challenging work, writing, creativity) that would not spark obvious (positive or negative) social evaluations in terms of morality in particular. In both the scientist and the novelist condition, participants were required to first make assessments for the typical scientist / novelist, before being asked to name three prominent personas from the respective occupations, for which they then repeated the same assessments. In both conditions, additional assessments were made for a typical citizen of one’s country (serving as a second control group; the original wording used in the questionnaire was “typical countryman”.) as well as for the self. Participants made judgments on the three stereotype content dimensions of morality, sociability, and competence (however, for reasons of parsimony, with only one item each: moral, warm, competent) as well as on the five moral foundations (harm, justice, loyalty, authority, and purity [20]). Additionally, we included four statements regarding contentious science topics that spark controversies amongst the public [11]. These statements were intended as a way to get at participants’ own attitudes toward science as well as their perceptions of how scientists would look upon these controversies. Again, all responses were given on a Likert type scale ranging from 1 for strongly disagree to 7 for strongly agree.

### Results

#### Analytic strategy

We started by generating scales for binding moral foundations (loyalty, authority, and purity) and individualizing moral foundations (harm, justice). We also created a scale that reflected general agreement with the four science topics: namely, humans cause CO_2_ emissions, vaccinations cause autism (reverse-scored), GMOs are safe, and humans have developed through evolution. Higher scores reflected opinions more in line with scientific consensus. The internal reliabilities for each of these scales within each of the targets varied from .53 to .73. Internal reliabilities for each group separately as well as zero-order correlations for all variables can be found in the S3 and S4 Tables.

To test for differences in ratings between the typical scientist, novelist, citizen, and self, as well as the first-to-mind prominent scientist and first-to-mind prominent novelist, we took the same approach as in Study 2. First, we conducted univariate ANOVAs, which were followed up by Tukey HSD tests (see Table 2). Again, this procedure was run for both the individual items, as well as the scale totals. Then, we used linear regression to test the extent to which participants’ agreement with the controversial science topics was predictive of a) their perceived agreement of the typical scientist with those topics as well as b) their perceived morality, warmth, and competence of the typical scientists. Furthermore, we repeated the same regression models with a different predictor, namely a scientist/novelist-knowledge score that reflected the extent to which participants correctly listed three examples of prominent scientists or novelists, which could range from 0 to 6: For each of the three provided examples, participants either scored 0 (no scientist named), 1 (person named is science-affiliated, but prominent for other skills e.g., business person Elon Musk), or 2 (person named is clearly scientist, e.g., Stephen Hawking). The same logic was applied to the novelist condition. Additionally, we tested the extent to which this knowledge score was predictive of the participant’s own agreement with the controversially debated science topics.

**Table 2:** Means and standard deviations for all groups and results of ANOVA and Tukey HSD tests.

	ANOVA		Tukey HSD					
	F	η^2^	1. Scientist	2. Novelist	3. Citizen	4. Self	5. 1^st^ scientist	6. 1^st^ novelist
Moral	27.20***	.122	5.30 (±1.11)^3^^,^^4^	4.96 (±0.96)	4.93 (±1.21)*	5.98 (±0.92)***	5.60 (±1.28)	5.45 (±1.19)
Warm	27.91***	.125	4.07 (±1.25)^2^^,^^3^^,^^4^^,^^5^^,^^6^	4.83 (±1.01)***	5.03 (±1.11)***	5.61 (±1.25)***	4.90 (±1.53)***	5.21 (±1.27)***
Competent	53.28***	.214	6.35 (±0.79)^2^^,^^3^	5.96 (±0.75)**	5.13 (±1.15)***	6.14 (±0.93)	6.46 (±0.98)	6.24 (±0.82)
Ind. foundations (F)	30.93***	.137	4.46 (±1.30)^2^^,^^3^^,^^4^^,^^5^^,^^6^	5.16 (±1.00)***	5.15 (±1.21)***	5.94 (±1.16)***	5.15 (±1.28)***	5.66 (±1.10)***
Harm/care	32.75***	.143	4.21 (±1.41)^2^^,^^3^^,^^4^^,^^5^^,^^6^	4.99 (±1.09)***	5.09 (±1.27)***	5.89 (±1.27)***	5.14 (±1.33)***	5.55 (±1.16)***
Justice/fairness	19.46***	.090	4.71 (±1.51)^2^^,^^3^^,^^4^^,^^6^	5.32 (±1.13)**	5.23 (±1.38)**	6.00 (±1.37)***	5.15 (±1.47)	5.77 (±1.24)***
Bind. foundations (F)	23.00***	.105	3.95 (±1.14)^3^	4.04 (±1.01)	5.02 (±0.94)***	4.15 (±1.57)	3.95 (±1.33)	4.12 (±1.24)
Loyalty	12.08***	.058	4.26 (± 1.25)^3^	4.42 (±1.16)	5.21 (±1.21)***	4.30 (±1.91)	4.54 (±1.52)	4.52 (±1.52)
Authority	21.28***	.098	3.98 (±1.62)^3^	3.83 (±1.37)	5.05 (±1.24)***	4.22 (±1.87)	3.60 (±1.71)	4.00 (±1.50)
Purity	12.21***	.059	3.69 (±1.75)^3^	3.98 (±1.45)	4.83 (±1.31)***	3.95 (±2.14)	3.79 (±1.71)	3.85 (±1.70)
Science topics (F)	26.80***	.120	5.90 (±0.88)^2^^,^^3^^,^^4^^,^^5^^,^^6^	4.97 (±0.78)***	4.73 (±0.87)***	5.27 (±1.39)***	5.53 (±1.08)*	4.98 (±1.04)***
Human CO_2_	9.08***	.044	5.96 (±1.19)^2^^,^^3^^,^^5^^,^^6^	5.34 (±1.08)**	5.03 (±1.14)***	5.62 (±1.75)	5.45 (±1.62)*	5.34 (±1.45)**
Vaccinations	12.12***	.058	4.94 (±1.43)^2^^,^^3^^,^^6^	4.29 (±1.33)***	4.00 (±1.38)***	4.73 (±1.73)	4.76 (±1.55)	4.45 (±1.36)*
GMO safety	21.57***	.099	5.47 (±1.22)^2^^,^^3^^,^^4^^,^^5^^,^^6^	4.06 (±1.23)***	4.18 (±1.34)***	4.33 (±1.88)***	4.92 (±1.43)*	3.97 (±1.35)***
Evolution	21.94***	.101	6.18 (±1.07)^2^^,^^3^^,^^4^^,^^6^	5.18 (±1.18)***	4.69 (±1.40)***	5.40 (±1.92)***	5.89 (±1.43)	5.18 (±1.50)***

*Note*. ANOVA degrees of freedom: 5, 978. Condition 1 and 5: *n* = 128, condition 2 and 6: *n* = 119, condition 3 and 4: *n* = 247. Subscripts refer to significant differences compared to

^2^typical novelist

^3^the typical citizen

^4^the self

^5^the first named prominent scientist, and

^6^the first named prominent novelist.

*p < .05

**p < .01

***p < .001

### Data analysis

All univariate ANOVAs were significant and are reported in Table 2 along with means and standard deviations for each item and scale. Tukey HSD tests showed that the typical scientist was perceived as more competent compared to both the typical novelist (*p* = .007, *d* = 0.506) and typical citizen (*p* < .001, *d* = 1.237). In terms of warmth, the results were mixed. Again, scientists were perceived as less sociable than both control groups (*ps* < .001, *d*_*novelist*_ = 0.669, *d*_*citizen*_ = 0.812). However, in terms of morality, a more complex picture emerges. Although scientists scored (marginally) higher on morality as a dimension of stereotype content compared to novelists (*p* = .090, *d* = 0.328) and citizens (*p* = .020, *d* = 0.319), they scored lower on almost all moral foundations. On the scale level, the typical scientist was perceived as less motivated to abide to individualizing moral foundations compared to the typical novelist (*p* < .001, *d* = 0.604) and citizen (*p* < .001, *d* = 0.549)–a finding that holds for both individual level foundations of harm/care (*ps* < .001, *d*_*novelist*_ = 0.619, *d*_*citizen*_ = 0.656) and justice/fairness (*p*_*novelist*_ = .008, *d*_*novelist*_ = 0.457; *p*_*citizen*_ = .010, *d*_*citizen*_ = 0.359). Similarly, the typical scientist was rated lower on binding moral foundations compared to the typical citizen (*p* < .001, *d* = 1.024), yet not compared to the typical novelist (*p* = .993, *d* = 0.084). For comparisons between the typical scientist and typical citizen, this pattern also holds on the individual level for all three binding moral foundations of loyalty (*p* < .001, *d* = 0.772), authority (*p* < .001, *d* = 0.742), and purity (*p* < .001, *d* = 0.738). The typical scientist’s agreement with controversially debated science topics was judged to be higher on all four topics than the typical novelist’s or citizen’s agreement, both on the scale (*p*_*novelist*_ < .001, *d*_*novelist*_ = 1.118; *p*_*citizen*_ < .001, *d*_*citizen*_ = 1.337) as well as on the individual item level (all *ps* < .001, *ds* > 0.546). Additionally, participants also completed ratings for themselves. In line with research on self-enhancement [37], participants generally rated themselves as more positive compared to the other groups, especially in terms of morality and warmth. Specifically, they perceived themselves to be more moral (*ps* < .001, *ds* > 0.667) and warmer (*ps* < .001, *ds* > 0.932) than all other groups. In terms of competence, they perceived themselves as more competent than the typical citizen (*p* < .001, *d* = 0.968) and half-way between the highly competent scientist (*p* = .221, *d* = 0.243) and the somewhat competent novelist (*p* = .477, *d* = 0.213). With regard to the moral foundations, participants rated themselves as more motivated to comply with individualizing moral foundations than all other groups (*ps* < .001, *ds* > 0.667). However, for binding moral foundations, participants self-evaluations scored closer to the lowest-scoring typical scientist (*p* = .691, *d* = 0.146) then to the highest-scoring typical citizen (*p* < .001, *d* = 0.672).

In the second part of Study 2, we found that the most frequently mentioned prominent scientists were all physicists: Albert Einstein, followed by Stephen Hawking, and Marie Curie. In comparison to the novelist condition, participants in the scientist condition generated fewer unique examples (69 scientists compared to 148 novelists) and were also less like to generate correct examples: only 53.91% of the participants were able to list three correct examples of prominent scientists, compared to 88.23% in the novelist condition (although this difference almost vanishes when only looking at the first-to-mind example: 87.50% correct for scientists, 91.60% for novelists).

In general, for both the scientist and novelist condition, the prominent examples that participants generated were rated more positively than their typical counterparts. Specifically, in comparison to the typical scientist, first-to-mind scientists were rated as significantly more sociable (*p* < .001, *d* = 0.594) and as more motivated to follow the moral foundation of harm/care (*p* < .001, *d* = 0.679). In terms of controversially debated science topics, the first-to-mind scientists tended to be somewhat more skeptical (i.e., less in line with scientific evidence) than typical scientists (*p* = .023, *d* = 0.376; especially on the human CO_2_ statement: *p* = .021, *d* = 0.359; and GMO safety statements: *p* = .023, *d* = 0.414), thus rendering them more similar in ratings to typical novelists, citizens, and the self. When making comparisons between perceptions of the typical novelist and the first-to-mind novelist, fewer differences surfaced as compared to the scientist condition.

Further, we explored whether the degree to which participants believed scientists to agree with the statements on controversially debated science topics could be predicted by their own agreement with these topics. Indeed, for each topic, participants’ own agreement was highly predictive of their perceived agreement of scientists with that statement (human CO_2_: *b* = .276, *t*(125) = 3.514, *p* < .001; vaccinations: *b* = .477, *t*(125) = 5.835, *p* < .001; GMO safety: *b* = .362, *t*(125) = 3.604, *p* < .001; evolution: *b* = .394, *t*(125) = 4.068, *p* < .001). Moreover, participants’ agreement with the science statements was also predictive of their perception of scientists in terms of morality, sociability, and competence. The extent to which participants agreed with the theory of evolution positively predicted their perceived morality of scientists, *b* = .258, *t*(122) = 2.695, *p* = .008. The extent to which participants believed in anthropogenic climate change predicted their perceived sociability of scientists, *b* = .200, *t*(122) = 2.008, *p* = .047. And last, participants’ perceptions of scientists’ competence was predicted by their agreement with anthropogenic climate change (*b* = .281, *t*(122) = 2.954, *p* = .004), the extent to which they disagreed with vaccinations causing autism (*b* = .220, *t*(122) = 2.315, *p* = .022) and the extent to which they did not agree with GMOs being safe (*b* = -.220, *t*(122) = -2.211, *p* = .029). (The predictive relations between participants’ agreement with controversially debated science topics and their perceived agreement of scientists with those topics as well as their perceived morality, warmth and competence of scientists also hold on the scale level, that is, when combining participants’ agreement with the four statements on controversially debated science topics into one score.)

Next, we tested the same models with a different predictor, namely the extent to which participants were able to correctly name three prominent examples of scientists. On average, participants achieved a scientist-knowledge score for the three prominent examples of 5.35 (out of 6, *SD* = 0.81). This scientist-knowledge score positively predicted participants’ perceived agreement of the typical scientist with the CO_2_ statement (*b* = .269, *t*(126) = 3.137, *p* = .002) and of the conception that vaccinations do not cause autism (*b* = .228, *t*(126) = 2.624, *p* = .009). For the typical scientist’s perceived morality, no effects of the scientist-knowledge score were observed. Lastly, we also tested whether participants’ scientist-knowledge score was predictive of their own agreement with the statements on controversially debated science topics. Indeed, their scientist-knowledge score positively predicted participants’ agreement with the conception that human emissions cause CO_2_ (*b* = .349, *t*(125) = 4.166, *p* < .001) as well as their agreement with the theory of evolution (*b* = .197, *t*(125) = 2.241, *p* = .027).

### Discussion

To a large extent, Study 2 replicates the findings of Study 1. As in Study 1, participants perceived scientists as less sociable (here, warm) and more competent compared to control groups (i.e., typical novelist or citizen). In addition, Study 2 yielded mixed findings on morality, revealing that while scientists tend to be perceived as moral when assessing morality along the three-dimensional approach to stereotype content, they seem to be perceived as comparatively immoral in terms of their compliance with the five moral foundations. Interestingly, while Study 1, in line with previous research [19], found associations between scientists and immorality to only hold for binding moral foundations, the results of Study 2 suggest that scientists also score significantly lower on individualizing moral foundations compared to the control groups. In fact, while within-comparisons show that scientists are indeed still perceived as less moral in terms of binding as opposed to individualizing moral foundations, similar comparisons within the control groups indicate that this difference is actually smaller for scientists as compared to novelists, typical citizens, or even the self. This results in larger between-group differences for individualizing as opposed to binding moral foundations between scientists and control groups, which–contrary to previous findings–may suggest that it is the relative neglect of individualizing foundations which accounts for more negative attitudes toward scientists as compared to control groups. This may especially be the case given that binding moral foundations seem to be less valued than individualizing foundations in general, as suggested by a) the lack of self-enhancement in the self-ratings as compared to ratings on the typical citizen, b) low correlations between binding moral foundations and positive dimensions such as sociability and competence (see Supplementary Materials), and c) Study 1’s positively perceived university-scientists only differing from the generally negatively perceived industry-scientist in terms of individualizing, but not binding moral foundations. Thus, even relatively small breaches against individualizing moral foundations may lead to substantial negative perceptions of scientists.

Similar to Study 1, Study 2 also investigated the effect that scientist framing has on stereotypical perceptions of scientists. In Study 2, participants not only made judgments about the typical scientist, but also about the first prominent scientist that came to their mind. Those first-to-mind scientists were overall rated more positively than the typical scientist, especially in terms of sociability and individualizing moral foundations (that is, where perceptions of typical scientists are most negative); a finding which also emerged comparing the first-to-mind and typical novelists. This could reflect a general effect of rating prominent examples instead of the prototype of an occupational group, as prominent examples are usually prominent for their positive impact on the world. Yet, it is interesting to note that fewer differences surfaced in the novelist condition as opposed to the scientist condition, indicating that there must be a scientist-specific effect. Combining this with the finding that participants had a harder time coming up with three correct examples of scientists as compared to novelists, and also mentioned fewer unique examples, our findings may suggest that participants’ attitudes toward the typical scientist are more heavily influenced by behavior that participants consider *possible* instead of what is *probable* (as low familiarity with scientists may not allow for an estimate of what is probable). In addition, attitudes toward typical scientists may be more heavily influenced by fictional scientist characters (e.g., cultural archetypes of evil scientists, such as Mary Shelley’s Dr. Frankenstein [38]), which–as we know from existing work on the image of scientists in pop-culture–are often portrayed as mad and dangerous, due to their unhealthy ambition in the pursuit of knowledge [9, 22, 23]. Empirical support for the idea that these cultural images of scientists may influence stereotypical perceptions of scientists comes from previous research showing that scientists are perceived as willing to prioritize knowledge gain over doing “the right thing” and thus are potentially more dangerous [19].

Lastly, Study 2 showed that the extent to which people are skeptical about science predicts their perceptions of scientists with regards to morality, warmth, competence, and the perceived standpoint of scientists on such controversies. Even more interesting though, the extent to which participants were able to correctly name three prominent scientists was also predictive of the perceived agreement of scientists as well as their own agreement with the controversial science topics. This suggests that an individual’s personal familiarity with scientists may be associated with their attitudes about science in general–an idea that resonates with recent suggestions that a person’s psychological distance to science (here, probably particularly perceived social distance) may impact their skepticism toward specific scientific debates or science in general [39] (see also [40, 41] for research on psychological distance and climate change denial).

## General discussion

In two studies, we explored stereotypical perceptions of scientists, with a particular focus on perceptions of morality. Together, these studies provide a number of insights. First, we found that when it comes to morality, findings seem to depend on the conceptualization of morality as well as its operationalization.

More specifically, we asked participants to evaluate scientists in terms of their morality along with sociability and competence (following the three-dimensional approach to stereotype content [29, 30]) as well as their compliance with the five moral foundations (following Moral Foundations Theory [20]). When morality was assessed along with sociability as part of the warmth dimension, scientists were generally perceived as highly moral (which counters previously established associations between scientists and immorality [19]) and low in sociability (which is in line with previous work [19, 27]). This stresses the importance of distinguishing between the two components of warmth. However, when assessing morality along the more pluralist lines of Moral Foundations Theory, scientists generally scored low on all five–but particularly the binding–moral foundations.

These contradictory findings can be somewhat reconciled with the perspective that morality within the three-dimensional stereotype content approach mostly captures the justice/fairness foundation, which is one of the two individualizing foundations on which scientists tend to score higher. Yet, our findings from Study 2 suggest that the difference between perceptions of scientists and various comparison groups in terms of morality is actually bigger for individualizing as opposed to binding moral norms, indicating that it may actually be the relative neglect of individualizing moral norms that accounts for negative perceptions of scientists. Support for this idea can also be found in Study 1, where the positively perceived university scientist differed from the negatively perceived industry scientist more in terms of individualizing than binding moral foundations. Thus, violations against binding moral foundations may weigh less in shaping negative attitudes toward scientists.

Clearly, more research is needed to disentangle the precise mechanisms and effects at play here. For now, we can sum up that our findings reflect the previously reported ambiguous public image of scientists. While on the one hand, people seem to trust scientists [26], on the other hand, people seem to perceive scientists as violating moral norms, an observation that–in contrast to previous work [19]–we made for both binding and individualizing foundations. A potential explanation for this apparent ambivalence could be found in taking context into account–people may be prone to put trust into scientists’ work behavior, yet not their personal behavior. This would be in line with the observed high scores in competence (people trust scientists’ professional competence) and low scores in sociability (people do not trust scientists’ social skills). Future research is necessary to identify the contextual situations and specific qualities in which people trust and mistrust scientists.

Another explanation for reconciling ambivalent findings could be found in different framings of science and scientists. We found that perceptions of scientists were heavily influenced by framing scientists as either working for a university or in the pharmaceutical industry, a differentiation that shows some overlap with McCright and colleagues’ [34] differentiation between impact and production science. Interestingly, perceptions of the typical scientists were more similar to perceptions of a scientist working in the pharmaceutical industry (i.e., doing production science), indicating that people seem to generally conceptualize scientists more as commercially working people who pursue economic benefits than employees of the public sector pursuing knowledge. This idea finds support in the divergent curiosity and desire perceptions of the two types of scientists. While university-affiliated scientists were perceived as highly motivated to follow their curiosity and less so to satisfy their desire, industry-affiliated scientists were perceived as highly motivated to satisfy their own desire and less so to follow their curiosity.

### Limitations

There are several limitations to the reported studies. First, as in any research studying attitudes towards specific groups, the choice of reference or comparison groups has an impact on the interpretation of results. No obvious comparison group exists for scientists, making the selection of appropriate control groups a difficult task. In the above studies, we tried to overcome this issue by comparing scientists with a variety of different groups, paying attention to selecting both generally positively perceived (religious people) as well as negatively perceived groups (atheists), in line with previous work [36]. Moreover, we also included comparisons with more neutral control groups (novelists, typical citizens), the self, and even comparisons within different types of scientists. Thereby, we aimed to capture a reliable picture of how scientists are perceived as opposed to how they compare to a specific group.

Second, a central finding in Study 1 was the divergent perceptions of university-affiliated as opposed to industry-affiliated scientists, with the latter generally perceived more negatively than the former. However, it is important to note that our Study 1 sample consisted of a convenience sample of university students. Therefore, it is possible that demand characteristics or mere exposure effects played a role in the more positive attitudes toward university-affiliated scientists. Although we addressed the limitation of using a convenience student sample by sampling through Amazon Mechanical Turk in Study 2, the specific finding on distinct perceptions of university- as opposed to industry-affiliated scientists warrants replication in a representative sample.

Third, in our research design, we did not specifically control for how perceptions of scientists may depend on the perceived gender of the scientist in question. In Study 1, only male personas were introduced through our stimulus material, thus preventing gender from acting as a confounding variable, yet at the same time neglecting it as a possible moderator. By using a bottom-up approach in which participants generated examples of scientists themselves, we moved beyond this limitation in Study 2. Yet, we found that most participants still thought of male as opposed to female scientists–only 8.5% of the generated examples were female. These findings are in line with years of results obtained with *draw-a-scientist* experiments [42, 43]. Thus, findings from Study 2 still largely reflect perceptions of male as opposed to female scientists–an issue that may have even been exaggerated by our unfortunate use of gender-biased language in one of the comparison groups (“typical countryman”). Thus, future research should play close attention to using gender-neutral language and systematically test how perceptions of scientists interact with gender perceptions.

### Implications

Despite legitimate limitations of the present research, our findings have implications for a range of research lines. First, they hold important insights for morality research in general, in showing that the conceptualization and operationalization of morality has serious consequences for the findings one can expect to obtain–to the extent that one might report opposite findings depending on the morality measure in question. Existing research seems to often overlook this ambiguity in the construct of morality. Second, our findings inform general research on stereotyping by highlighting the importance of differentiating between the sociability and morality components in the warmth factor of the SCM. Until today, much research on stereotyping continues to neglect this differentiation of warmth [44–46], which has serious ramifications for research on groups that are perceived as high in morality and low in sociability—such as scientists—or the other way around.

Most importantly, our findings provide insights into attitudes toward scientists. First, they suggest that framing effects may be able to explain and account for some ambivalent or contradictory findings within the current body of literature, as even slight differences in framings of scientists between studies may have triggered divergent perceptions. Second, these effects may not only play a role in attitudes toward scientists, but also toward science in general. The type of science in question (production vs impact science) should thus be taken into consideration when assessing potential drivers behind science skepticism. And lastly, the observed framing effects have important implications for science communication. As we found people to hold more negative attitudes about scientists working in industry as opposed to scientists working in academia, it may be advisable to emphasize scientists’ university backgrounds when they communicate with the public. This may be particularly important whenever there is an association with pharmaceutical work (the example industry used in the present research). Highlighting work by university-affiliated researchers or actively stressing certain qualities in the industry scientists (e.g., pursuing knowledge instead of financial gain) may help to shape more positive attitudes toward the respective scientist. Given the observed relations between attitudes toward scientists and skepticism toward science in general, this may then help to decrease overall skepticism toward the integrity of the presented scientific findings–an objective of high importance in light of the problem of public repudiation of science.

## Supporting information

S1 Table Cronbach Alpha’s and Pearson correlations for generated scales in Study 1. (DOCX)

S2 Table Zero-order correlations of variables introduced in Study 1. (DOCX)

S3 Table Cronbach Alpha’s and Pearson correlations for generated scales in Study 2. (DOCX)

S4 Table Zero-order correlations of variables introduced in Study 2. (DOCX)
