# Supplementary material for: The (im-)moral scientist? Measurement and framing effects shape the association between scientists and immorality
Source: PLoS One. 2022 Oct 3;17(10):e0274379. doi: 10.1371/journal.pone.0274379 (PMC9529126; doi:10.1371/journal.pone.0274379)
Supplement: S1 Table — (DOCX) [file pone.0274379.s001.docx]

**S1. Supplementary Table 1**

***Cronbach Alpha’s and Pearson Correlations for Generated Scales in Study 1***

|  | Total | Scientist | Atheist | Religious | University-S | Pharma-S |
| --- | --- | --- | --- | --- | --- | --- |
| Morality (F) | 0.88 | 0.87 | 0.81 | 0.82 | 0.87 | 0.92 |
| Sociability (F) | 0.91 | 0.90 | 0.90 | 0.90 | 0.89 | 0.92 |
| Competence (F) | 0.91 | 0.90 | 0.82 | 0.85 | 0.84 | 0.77 |
| Immoral character (F) | 0.74 | 0.72 | 0.64 | 0.61 | 0.90 | 0.82 |
| Ind. foundations (F) | 0.58 | n/a | n/a | n/a | 0.34 | 0.57 |
| Bind. foundations (F) | 0.57 | n/a | n/a | n/a | 0.53 | 0.63 |

*Note*. *N* = 140
