# Supplementary material for: The (im-)moral scientist? Measurement and framing effects shape the association between scientists and immorality
Source: PLoS One. 2022 Oct 3;17(10):e0274379. doi: 10.1371/journal.pone.0274379 (PMC9529126; doi:10.1371/journal.pone.0274379)
Supplement: S2 Table — (DOCX) [file pone.0274379.s002.docx]

**S2. Supplementary Table 2**

|  | 1. | 1.1. | 1.2. | 1.3. | 2. | 2.1. | 2.2. | 2.3. | 3. | 3.1. | 3.2. | 3.3. | 4. | 4.1. | 4.2. | 5. | 5.1. | 5.2. | 6. | 6.1. | 6.2. | 6.3. | 7. | 8. |
| --- | --- | --- | --- | --- | --- | --- | --- | --- | --- | --- | --- | --- | --- | --- | --- | --- | --- | --- | --- | --- | --- | --- | --- | --- |
| 1. Morality (F) | **1** | - | - | - | - | - | - | - | - | - | - | - | - | - | - | - | - | - | - | - | - | - | - | - |
| 1.1. Honest | **.90** | **1** | - | - | - | - | - | - | - | - | - | - | - | - | - | - | - | - | - | - | - | - | - | - |
| 1.2. Sincere | **.90** | **.72** | **1** | - | - | - | - | - | - | - | - | - | - | - | - | - | - | - | - | - | - | - | - | - |
| 1.3. Trustworthy | **.89** | **.71** | **.69** | **1** | - | - | - | - | - | - | - | - | - | - | - | - | - | - | - | - | - | - | - | - |
| 2. Sociability (F) | **.66** | **.60** | **.61** | **.57** | **1** | - | - | - | - | - | - | - | - | - | - | - | - | - | - | - | - | - | - | - |
| 2.1. Likeable | **.61** | **.57** | **.57** | **.51** | **.93** | **1** | - | - | - | - | - | - | - | - | - | - | - | - | - | - | - | - | - | - |
| 2.2. Warm | **.58** | **.52** | **.53** | **.50** | **.91** | **.75** | **1** | - | - | - | - | - | - | - | - | - | - | - | - | - | - | - | - | - |
| 2.3. Friendly | **.64** | **.57** | **.59** | **.57** | **.93** | **.82** | **.75** | **1** | - | - | - | - | - | - | - | - | - | - | - | - | - | - | - | - |
| 3. Competence (F) | **.46** | **.40** | **.41** | **.42** | **.31** | **.30** | **.26** | **.30** | **1** | - | - | - | - | - | - | - | - | - | - | - | - | - | - | - |
| 3.1. Competent | **.45** | **.39** | **.42** | **.41** | **.34** | **.32** | **.29** | **.32** | **.91** | **1** | - | - | - | - | - | - | - | - | - | - | - | - | - | - |
| 3.2. Intelligent | **.37** | **.34** | **.33** | **.34** | **.22** | **.23** | **.19** | **.21** | **.93** | **.76** | **1** | - | - | - | - | - | - | - | - | - | - | - | - | - |
| 3.3. Skillful | **.45** | **.39** | **.41** | **.42** | **.31** | **.30** | **.26** | **.31** | **.93** | **.76** | **.81** | **1** | - | - | - | - | - | - | - | - | - | - | - | - |
| 4. Immoral character (F) | **-.49** | **-.43** | **-.42** | **-.45** | **-.48** | **-.47** | **-.42** | **-.45** | **-.30** | **-.26** | **-.27** | **-.29** | **1** | - | - | - | - | - | - | - | - | - | - | - |
| 4.1. Bad | **-.46** | **-.42** | **-.40** | **-.42** | **-.46** | **-.44** | **-.40** | **-.42** | **-.26** | **-.21** | **-.23** | **-.28** | **.93** | **1** | - | - | - | - | - | - | - | - | - | - |
| 4.2. Bad standards | **-.44** | **-.39** | **-.39** | **-.42** | **-.44** | **-.44** | **-.37** | **-.41** | **-.29** | **-.26** | **-.27** | **-.27** | **.94** | **.74** | **1** | - | - | - | - | - | - | - | - | - |
| 5. Ind. foundations (F) | **.78** | **.72** | **.74** | **.76** | **.60** | **.56** | **.54** | **.59** | **.46** | **.43** | **.38** | **.38** | **-.56** | **-.53** | **-.56** | **1** | - | - | - | - | - | - | - | - |
| 5.1. Harm/care | **.63** | **.57** | **.58** | **.64** | **.56** | **.53** | **.49** | **.55** | **.34** | **.37** | **.24** | **.25** | **-.43** | **-.40** | **-.44** | **.89** | **1** | - | - | - | - | - | - | - |
| 5.2. Honesty | **.76** | **.71** | **.73** | **.72** | **.51** | **.46** | **.47** | **.50** | **.48** | **.39** | **.43** | **.43** | **-.56** | **-.54** | **-.55** | **.88** | **.57** | **1** | - | - | - | - | - | - |
| 6. Bind. foundations (F) | **.25** | **.20** | **.25** | **.26** | **.27** | **.19** | **.31** | **.27** | .15 | .14 | .14 | .12 | **-.21** | **-.20** | **-.20** | **.38** | **.30** | **.38** | **1** | - | - | - | - | - |
| 6.1. Loyalty | **.40** | **.35** | **.35** | **.44** | **.36** | **.28** | **.36** | **.36** | **.26** | **.24** | **.24** | **.19** | **-.27** | **-.26** | **-.26** | **.50** | **.42** | **.47** | **.68** | **1** | - | - | - | - |
| 6.2. Authority | -.07 | -.06 | -.06 | -.09 | .02 | -.05 | .09 | .02 | -.02 | .00 | -.03 | -.04 | .02 | .02 | .02 | .08 | .03 | .11 | **.72** | **.30** | **1** | - | - | - |
| 6.3. Purity | **.20** | .13 | **.22** | **.21** | .20 | .17 | **.20** | **.20** | .11 | .07 | .11 | .10 | **-.17** | **-.17** | **-.17** | **.26** | **.21** | **.25** | **.77** | **.25** | **.35** | **1** | - | - |
| 7. Curiosity | **.60** | **.60** | **.55** | **.56** | **.50** | **.51** | **.44** | **.46** | **.46** | **.36** | **.44** | **.41** | **-.44** | **-.43** | **-.43** | **.47** | **.35** | **.49** | .12 | .16 | -.08 | .16 | **1** | - |
| 8. Desire | -.11 | -.07 | -.15 | -.09 | -.11 | -.06 | -.12 | -.12 | .06 | .01 | .07 | .09 | **.19** | .14 | **.21** | -.15 | **-.20** | -.07 | -.05 | .03 | -.04 | -.09 | .07 | **1** |

***Zero-Order Correlations of Variables Introduced in Study 1.***

*Note*. *N* = 140. Correlations in bold are significant at α = .05.
