# Supplementary material for: The (im-)moral scientist? Measurement and framing effects shape the association between scientists and immorality
Source: PLoS One. 2022 Oct 3;17(10):e0274379. doi: 10.1371/journal.pone.0274379 (PMC9529126; doi:10.1371/journal.pone.0274379)
Supplement: S3 Table — (DOCX) [file pone.0274379.s003.docx]

**S3. Supplementary Table 3**

***Cronbach Alpha’s and Pearson Correlations for Generated Scales in Study 2***

|  | Total | Scientist | Novelist | Citizen | Self | 1^st^ scientist | 1^st^ novelist |
| --- | --- | --- | --- | --- | --- | --- | --- |
| Ind. foundations (F) | 0.70 | 0.57 | 0.61 | 0.61 | 0.70 | 0.73 | 0.70 |
| Bind. foundations (F) | 0.66 | 0.57 | 0.64 | 0.68 | 0.53 | 0.65 | 0.69 |
| Controversies (F) | 0.73 | 0.73 | 0.53 | 0.57 | 0.76 | 0.70 | 0.71 |

*Note*. *N* = 247
