# Supplementary material for: The (im-)moral scientist? Measurement and framing effects shape the association between scientists and immorality
Source: PLoS One. 2022 Oct 3;17(10):e0274379. doi: 10.1371/journal.pone.0274379 (PMC9529126; doi:10.1371/journal.pone.0274379)
Supplement: S4 Table — (DOCX) [file pone.0274379.s004.docx]

**S4. Supplementary Table 4**

|  | 1. | 2. | 3. | 4. | 4.1. | 4.2. | 5. | 5.1. | 5.2. | 5.3. | 6. | 6.1. | 6.2. | 6.3. | 6.4. |
| --- | --- | --- | --- | --- | --- | --- | --- | --- | --- | --- | --- | --- | --- | --- | --- |
| 1. Moral | **1** | - | - | - | - | - | - | - | - | - | - | - | - | - | - |
| 2. Warm | **.56** | **1** | - | - | - | - | - | - | - | - | - | - | - | - | - |
| 3. Competent | **.54** | **.31** | **1** | - | - | - | - | - | - | - | - | - | - | - | - |
| 4. Ind. foundations (F) | **.54** | **.55** | **.32** | **1** | - | - | - | - | - | - | - | - | - | - | - |
| 4.1. Harm/care | **.53** | **.58** | **.30** | **.90** | **1** | - | - | - | - | - | - | - | - | - | - |
| 4.2. Justice/fairness | **.46** | **.43** | **.28** | **.91** | **.66** | **1** | - | - | - | - | - | - | - | - | - |
| 5. Bind. foundations (F) | **.12** | **.21** | -.06 | .03 | **.07** | .00 | **1** | - | - | - | - | - | - | - | - |
| 5.1. Loyalty | **.14** | **.19** | -.01 | .06 | **.07** | .03 | **.79** | **1** | - | - | - | - | - | - | - |
| 5.2. Authority | **.10** | **.19** | **-.07** | .03 | .06 | .01 | **.81** | **.53** | **1** | - | - | - | - | - | - |
| 5.3. Purity | **.06** | **.13** | -.06 | -.01 | .03 | -.04 | **.77** | **.38** | **.41** | **1** | - | - | - | - | - |
| 6. Science topics (F) | **.18** | .03 | **.26** | **.19** | **.13** | **.21** | **-.41** | **-.24** | **-.31** | **-.40** | **1** | - | - | - | - |
| 6.1. Human CO_2_ | **.23** | **.14** | **.24** | **.31** | **.25** | **.31** | **-.30** | **-.20** | **-.23** | **-.27** | **.75** | **1** | - | - | - |
| 6.2. Vaccinations | **.11** | .00 | **-.16** | **.14** | **.12** | **.14** | **-.32** | **-.19** | **-.25** | **-.31** | **.72** | **.43** | **1** | - | - |
| 6.3. GMO safety | **.11** | -.02 | **.14** | .01 | -.02 | .03 | **.20** | **-.10** | **-.15** | **-.21** | **.73** | **.35** | **.35** | **1** | - |
| 6.4. Evolution | **.08** | -.02 | **.22** | .**12** | .05 | **.15** | **-.40** | **-.23** | **-.30** | **-.39** | **.77** | **.48** | **.36** | **.43** | **1** |

***Zero-Order Correlations of Variables Introduced in Study 2.***

*Note*. *N* = 247. Correlations in bold are significant at α = .05.
